# Lectotypification and Morphological Re-Evaluation of *Arthopyrenia molinii* Beltr. Based on Historical Material and Comparison with Related Species

**DOI:** 10.3390/plants15213431

**Published:** 2026-11-04

**Authors:** Deborah Isocrono, Sonia Ravera

**Affiliations:** 1 Department of Agricultural, Forest and Food Sciences (DISAFA), University of Torino, Largo Paolo Braccini 2, 10095 Grugliasco, Italy; deborah.isocrono@unito.it; 2 Department of Biological, Chemical and Pharmaceutical Sciences and Technologies (STeBiCeF), University of Palermo, Via Archirafi 38, 90123 Palermo, Italy

**Keywords:** Dothideomycetes, historical taxonomy, perithecioid ascomata

## Abstract

*Arthopyrenia molinii*, described from northern Italy in 1858, has never been formally typified or critically reassessed. In the present study, historical collections preserved in European herbaria were examined to locate and evaluate the original material of the species. A specimen preserved in the A.B. Massalongo Herbarium (VER) is designated here as the lectotype, and *A. molinii* is redescribed on the basis of the lectotype and additional historical material, with emphasis on thallus, ascomatal, hamathecial and ascospore characters. The species is compared with morphologically similar European taxa, particularly *A. parolinii*, *A. platypyrenia*, and the pluriseptate varieties of *A. persoonii* historically treated as *A. grisea*. *Arthopyrenia molinii* is characterized by a persistent epiphloeodal tartareous thallus, relatively large perithecia frequently associated with dark areoles, and narrowly clavate to obclavate, 5–7-septate ascospores. This study establishes a nomenclatural reference for *A. molinii* and provides a provisional morphological circumscription based on historical material. *A. molinii* appears morphologically distinguishable from the other pluriseptate taxa examined here; however, because it has not been recollected in recent decades its intraspecific variation and the stability of the diagnostic characters identified cannot be adequately assessed.

## 1. Introduction

The genus *Arthopyrenia* A. Massal. poses challenges in both generic circumscription and species delimitation. Historically, the name has been applied broadly to species with blackish perithecia and 1-to several septate hyaline ascospores. The genus includes both lichenized and non-lichenized species. The number of accepted species vary considerably depending on the generic concept adopted, reflecting the taxonomic instability of the group. Its taxonomic circumscription has undergone substantial changes in recent years. Phylogenetic studies have shown that the broad historical concept of *Arthopyrenia* encompassed several unrelated lineages within Trypetheliaceae, leading to the recognition of distinct genera for groups formerly included in *Arthopyrenia* sensu lato [1]. Molecular studies based on the type species, *A. cerasi* (Schrad.) A. Massal., placed *Arthopyrenia* within Trypetheliaceae and resulted in the synonymization of Arthopyreniaceae with Trypetheliaceae [2]. Consequently, the current circumscription of *Arthopyrenia* differs considerably from the broad historical concept of the genus, which included a heterogeneous assemblage of lichenized and non-lichenized fungi. Recent phylogenetic work further supports *Arthopyrenia* s.str. as a distinct lineage within Trypetheliaceae [3]. Despite these advances, numerous species historically referred to *Arthopyrenia* remain poorly understood, particularly those described in the nineteenth century and never critically reassessed [4].

This problem is particularly acute for several nineteenth-century European taxa names, including *A. molinii* Beltr., *A. parolinii* Beltr., and the pluriseptate varieties of *A. persoonii* Massal. subsequently associated with *A. grisea* (Schaer.) Körb. [5]. These taxa have not been recollected in recent decades and are known mainly from historical herbarium material. Some names of these taxa have been formally typified [6] or critically evaluated on the basis of historical material [7]. Nevertheless, their taxonomic interpretation remains unresolved, particularly in relation to the original descriptions, type material, and subsequent species concepts.

The historical treatment of these taxa also highlights the difficulty of assigning diagnostic weight to individual morphological characters. In species with perithecioid ascomata and pluriseptate ascospores, ascospore septation has frequently been used as an important comparative character. However, its significance has varied according to the taxonomic concept adopted and has generally been evaluated in combination with characters of the thallus, ascomata, hamathecium, asci, and ascospores. This variation in the relative diagnostic weight assigned to these characters contributed to the different species concepts applied to pluriseptate *Arthopyrenia*.

*Arthopyrenia molinii* was described by Francesco Beltramini de’ Casati in 1858 from Monte Summano and Angarano (Bassano del Grappa, Vicenza, northern Italy). The species has never been formally typified or critically re-examined since its original description, and its morphology has not been reassessed since its original description. Beltramini, a student and close associate of Abramo Bartolomeo Massalongo, dedicated much of his work to the lichen flora of the Bassano del Grappa area [8]. Material attributable to Beltramini is preserved in several European herbaria, including the A.B. Massalongo Herbarium (VER). The close historical association between Beltramini and Massalongo [8,9] helps explain the presence of Beltramini’s collections in the Massalongo herbarium and was therefore relevant to the search for original material.

The original description of *A. molinii* includes several potentially diagnostic features, notably an epiphloeodal thallus, perithecia frequently associated with dark areoles, and pluriseptate ascospores. The examination of the original material therefore provides an opportunity not only to establish the nomenclatural identity of *A. molinii*, but also to evaluate the combination of morphological characters that distinguishes it from historically associated taxa.

The present study aims to clarify the identity of *A. molinii* through the examination of historical material, to designate a lectotype, and to provide a detailed morphological account of the species. Its affinities are assessed through comparison with morphologically similar European taxa with particular attention to characters useful for species delimitation.

## 2. Results

### 2.1. Taxonomic Treatment of Arthopyrenia molinii

**Figure 1:**
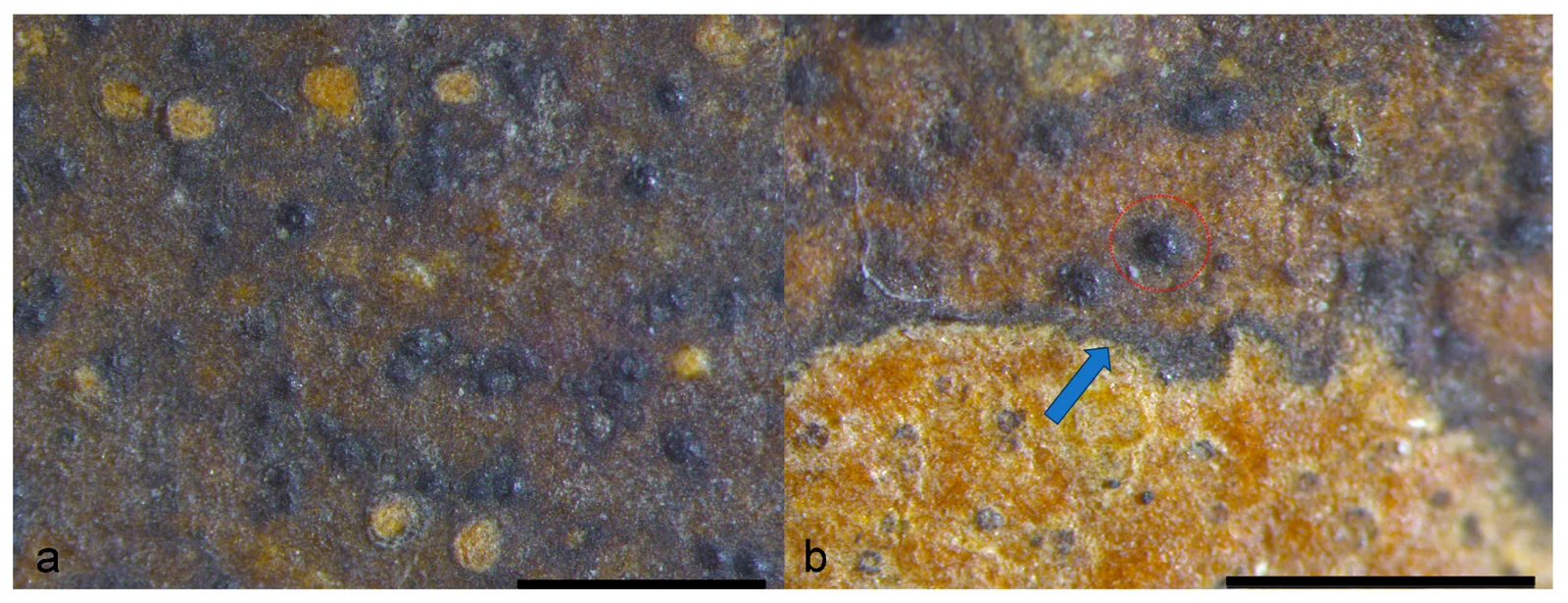
*Arthopyrenia molinii*, enlarged view of bark fragments of the exsiccatum from the A.B. Massalongo lichen herbarium VER (lectotype) (**a**) Part of the central bark fragment; (**b**) Part of the right-hand bark fragment of the exsiccatum: arrows indicate the black hypothalline line, and the circle highlights the dark areole surrounding the base of a perithecium. Scale bars = 1 mm.

*Arthopyrenia molinii* Beltr., Licheni Bassanesi: 240. 1858 MycoBank MB#377171.
≡ *Arthopyrenia summana* Beltr. in litt. ad Massal., Octob. 1857 (ined. nom. non val. publ.)
≡ *Spermatodium molinii* (Beltr.) Trevis., Conspectus Verruc.: 11. 1860.
Lectotype (designated here): ITALY. Veneto: province of Vicenza, Santorso at the foot of Monte Summano (“Campi del M. Summano a S. Orso”), on bark of *Fraxinus ornus* L. (“sugli orni”), n.d., F. Beltramini de’ Casati s.n., ex Herb. Beltramini [VER-AM4491! (Herb. A.B. Massalongo)].
Description:
Thallus: Non-lichenized, epiphloedic, very thin and continuous, dark brown to olive-brown, tartareous (Figure 1a), usually delimited by a black hypothalline line (Figure 1b).

**Figure 2:**
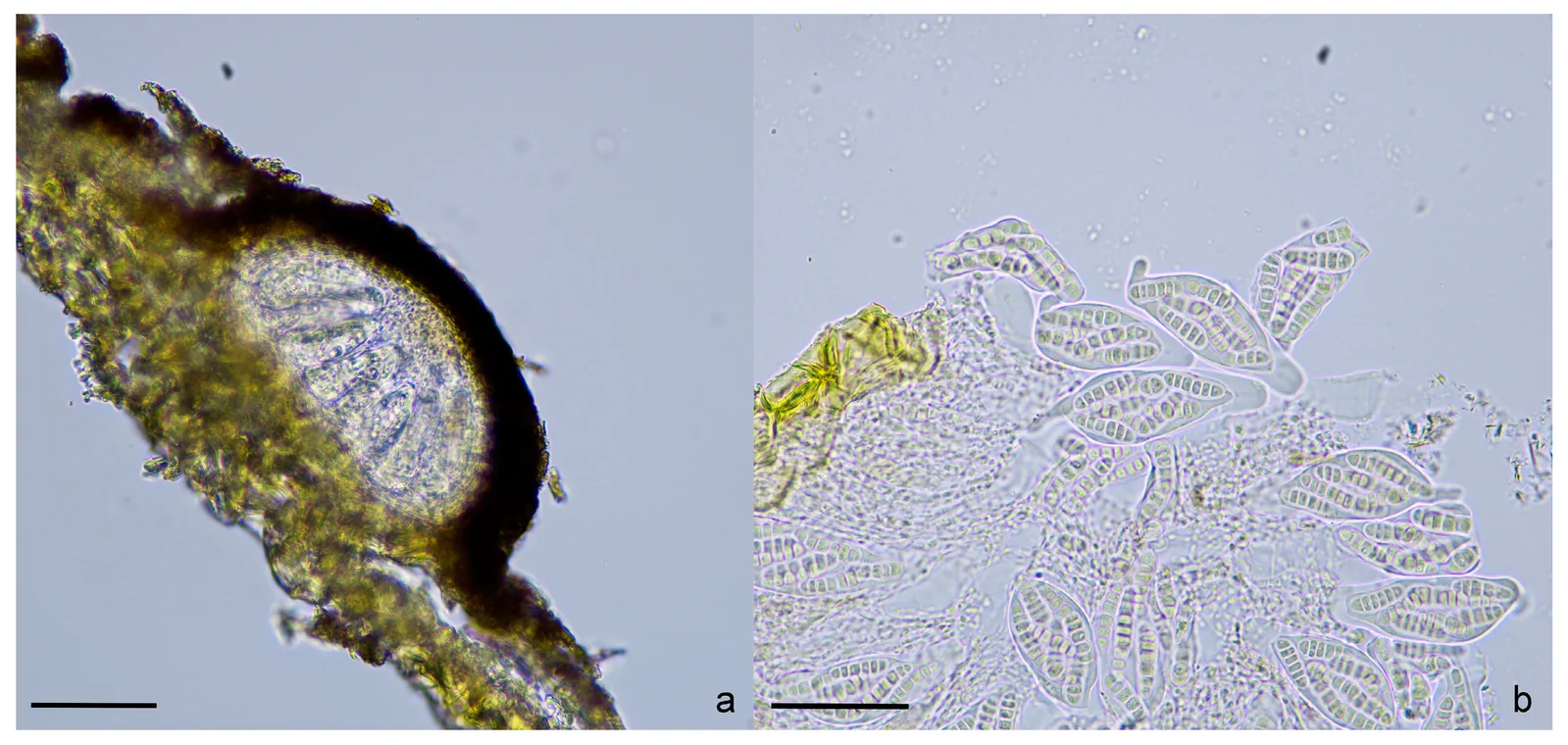
*Arthopyrenia molinii*. Historical exsiccatum preserved in RO. (**a**) Vertical section through a perithecium; (**b**) Bitunicate asci and pluriseptate ascospores in 10% KOH. Scale bars = 50 µm.

Ascomata: Perithecioid, black, 0.18–0.24 mm in diameter, subglobose to broadly ellipsoid in outline, superficial to semi-erumpent, usually surrounded at the base by a dark areola (Figure 1b); ostiolar region slightly flattened or depressed. Ascomatal wall 18–25 µm thick, dark brown, composed of textura intricata, discontinuous below the hamathecium; involucrellum clypeate, dark reddish brown, K−; exciple hyaline, poorly developed (Figure 2a). Hamathecium: Composed of moniliform pseudoparaphyses that dissolve, leaving only scattered fragments embedded in a gelatinous matrix; periphysoids absent. **Asci:** Bitunicate, 55–70 × 18–25 µm, cylindric-clavate, 8-spored, with a distinct, non-amyloid apical region (Figure 2b). Ascospores: Hyaline, 21–23 × 4–5 µm, 8 per ascus, irregularly arranged, narrowly clavate to obclavate with rounded apices, 5–7-septate, slightly constricted at the septa (appearing weakly didymoid at the median septum); perispore not evident. Pycnidia and Chemistry: Pycnidia not observed. Color tests negative (K−, I−).

In the protologue of *A. molinii* is provided a Latin diagnosis followed by a detailed description in Italian and illustrations [8]. The species was described as having a thin, blackish, epiphloedal thallus; numerous black perithecioid ascomata, referred to as apothecia by Beltramini, often surrounded at the base by a dark areole; clavate-ventricose, eight-spored asci lacking paraphyses; and linear-ellipsoid to subclavate, 6–8-locular ascospores, three to four times longer than wide.

Beltramini indicated the habitat as occurring “sugli orni nel monte Summano, nonchè in Angarano”, i.e., on *Fraxinus ornus* L. at Monte Summano and at Angarano (both near Bassano del Grappa, northern Italy). No holotype was explicitly designated in the protologue. Although the protologue includes illustrations, it does not identify a single specimen or gathering as the unequivocal basis of the description. Instead, Beltramini referred to material collected at two localities, Monte Summano and Angarano. Furthermore, the historical specimens examined bear no explicit collection date, and their labels do not provide sufficient evidence to establish that any single specimen was the only element used by Beltramini in preparing the original account. Based on the available evidence, therefore, no single specimen or illustration can be confidently recognized as the holotype, and lectotypification is necessary to establish a single nomenclatural reference for the name. The specimen preserved in VER is selected as the lectotype because its provenance from Beltramini’s herbarium is documented, it is the most complete specimen examined, its label provides precise locality information, and it corresponds closely to the protologue.

The epithet “Summana”, cited in the protologue as “in litteris ad Massal.” (Oct. 1857), represents an unpublished provisional name referring to Monte Summano. Beltramini subsequently validly published the species under the name *Arthopyrenia molinii*, honouring Raffaele Molin (1825–1887), a distinguished Italian naturalist and professor of Natural History at the University of Padua, where Beltramini studied.

Examination of historical collections revealed several potential specimens attributable to the original material of *A. molinii*, including material preserved in the A.B. Massalongo lichen herbarium (VER) that corresponds morphologically to the protologue.

**Figure 3:**
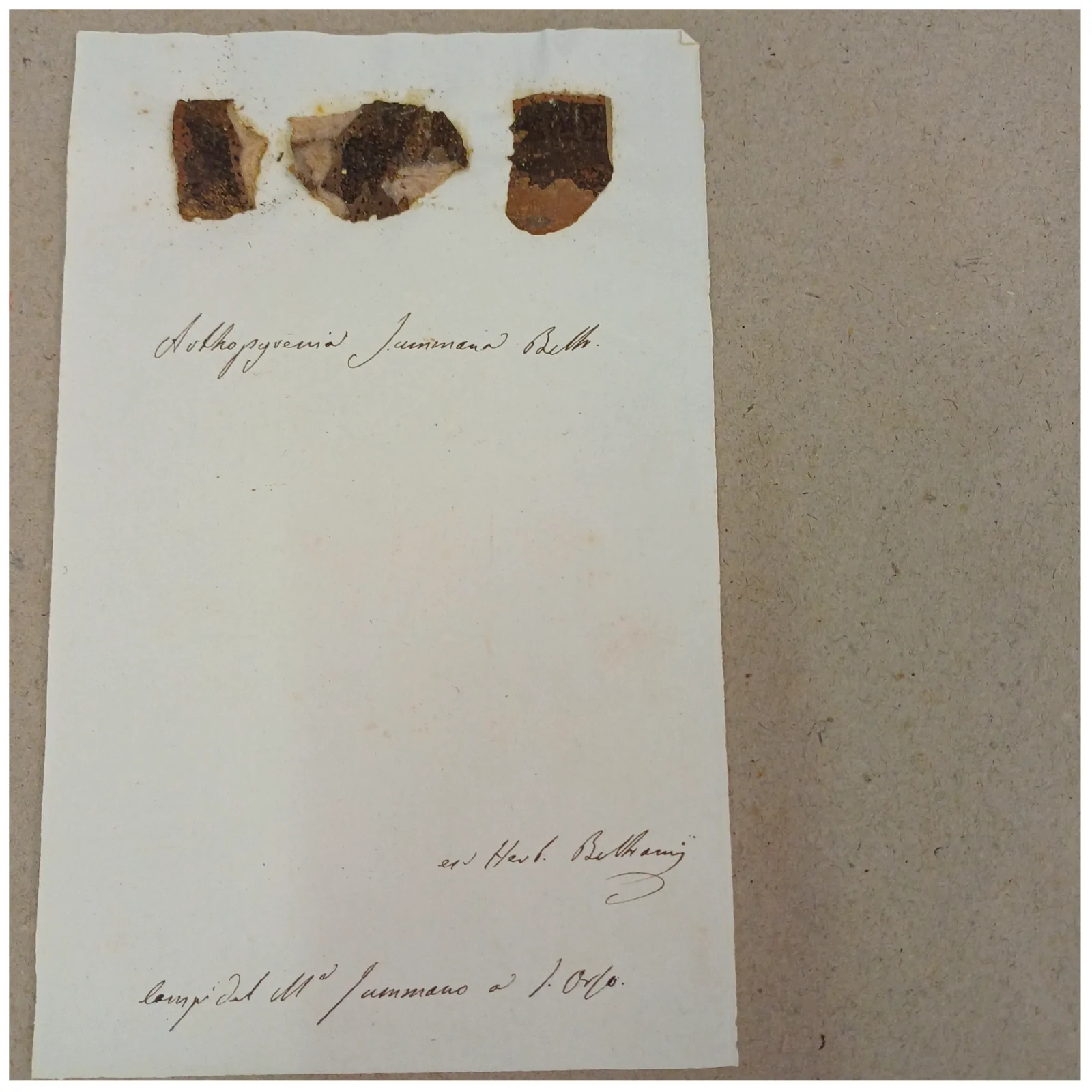
Exsiccata of *Arthopyrenia molinii* Beltramini preserved in the A.B. Massalongo Lichen Herbarium (VER) with the handwritten name *Arthopyrenia summana* Beltr., annotation “ex Herb. Beltramini”, and collection locality.

The specimen in VER consists of three bark fragments glued to the herbarium sheet (Figure 3). The label reports the name “*Arthopyrenia Summana* Beltr.”, the locality “Campi del M. Summano a S. Orso”, i.e., “fields of Monte Summano in Santorso”, thus providing a more precise collecting site than the one in the protologue. Santorso is a municipality located at the foot of Monte Summano. The annotation “ex Herb. Beltramini” indicates that the specimen now preserved at VER originated directly from Beltramini’s herbarium. This annotation, together with the other handwritten notes on the sheet, is in Massalongo’s handwriting, as confirmed by a calligraphic comparison with original material preserved in the Library of the University of Padua (see https://phaidra.cab.unipd.it/).

All examined sheets (Table A1) consistently include exsiccata labels from Lichenes rariores Veneti (no. 123), indicating “*Arthopyrenia molinii* Beltr., L. Bass. p. 240” and often the unpublished name “*Arthopyrenia summana* Beltr. in Herb. Mass.”. The locality is given as “ad *Fraxinus ornus* in prov. Vicentinae, M. Summano, Beltram.”, supporting their interpretation as material originating from Beltramini’s collection and subsequently distributed by Massalongo. Particularly noteworthy is the specimen preserved in M (M-0207337/630042/287563) originating from the Arnold herbarium (Ex Herb. Arnold, no. 504), which shows a different label layout and includes handwritten annotations attributable to Massalongo (including the species name credited to Beltramini and a number, presumably “86”). Additional handwritten notes by Massalongo occur on other sheets, including further material in M.

The examination of the lectotype and additional historical material supports the recognition of *A. molinii* as a morphologically distinctive taxon within the European pluriseptate *Arthopyrenia* complex. Rather than being defined by a single diagnostic feature, its identity is supported by a consistent combination of thallus, ascomatal, and ascospore characters. In particular, the persistent epiphloeodal and tartareous thallus, the relatively large perithecia frequently associated with dark basal areoles, and the narrowly clavate to narrowly obclavate, 5–7-septate ascospores provide the most useful combination for recognizing the species in the historical material examined here.

The comparison with related taxa also indicates that ascospore septation alone has limited diagnostic value within this group. Pluriseptate ascospores with more than 3 septa occur in several historically associated taxa, but their taxonomic significance can be assessed only in combination with other characters, particularly thallus structure, ascomata morphology, and ascospore shape and dimensions. The morphological distinctiveness of *A. molinii* therefore lies in the overall character combination rather than in the number of ascospore septa alone.

### 2.2. Morphological Comparison with Related European Taxa

European species of *Arthopyrenia* with pluriseptate ascospores remain insufficiently studied and have not been critically revised in recent literature. This situation partly reflects the difficulties associated with working on nineteenth-century material, which is often represented by only a few surviving specimens that may be difficult to trace or obtain on loan and, in many cases, known exclusively from the locus classicus [10].

Several European taxa are particularly relevant to the interpretation of *Arthopyrenia molinii*, particularly *A. parolinii*, *A. platypyrenia*, and the pluriseptate varieties of *A. persoonii* that Foucard [5] treated as conspecific with *A. grisea*. Although these taxa share some morphological features with *A. molinii*, especially the presence of pluriseptate ascospores, they differ in thallus, ascomatal, and ascospore characters.

#### 2.2.1. *Arthopyrenia parolinii* Beltr.

**Figure 4:**
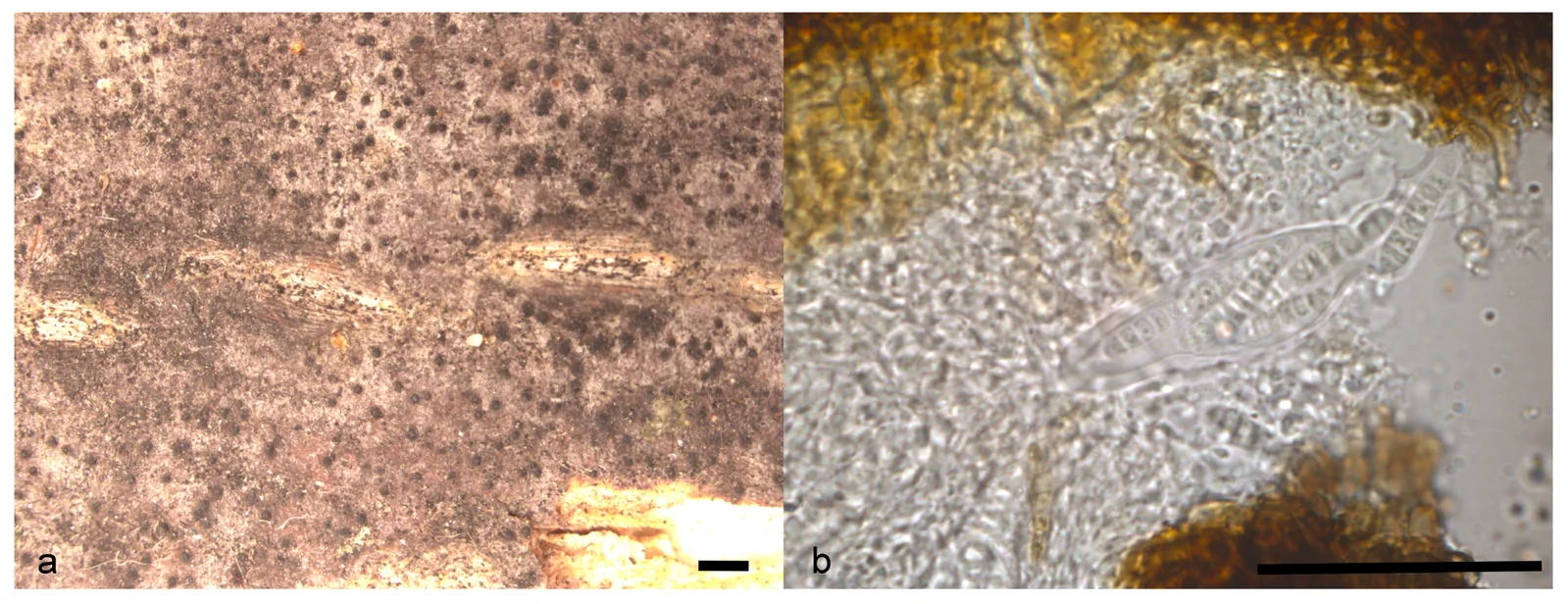
*Arthopyrenia parolinii*. (**a**) Thallus from the A.B. Massalongo lichen herbarium VER (lectotype). (**a**) Part of the central bark fragment, scale bar = 1 mm; (**b**) Bitunicate asci and pluriseptate ascospores in 10% KOH, scale bar = 50 µm.

*Arthopyrenia parolinii* was described together with *A. molinii* by Beltramini in his publication on the lichen flora of the Bassano del Grappa area [8]. It differs from *A. molinii* mainly in the thallus morphology and in the arrangement and morphology of the perithecia [7]. The thallus of *A. parolinii* is initially hypophloeodic, only later becoming epiphloeodic, thin, arachnoid and widely effuse, whereas in *A. molinii* it is consistently epiphloeodic and forms small, dark, tartareous patches. The perithecia of *A. parolinii* are smaller, uniformly scattered and mostly solitary, whereas those of *A. molinii* are often aggregated or locally confluent and commonly surrounded by a dark areole. In addition, the ascospores of *A. parolinii* are broadly clavate, whereas those of *A. molinii* are slenderer, narrowly clavate to narrowly obclavate (Figure 4).

#### 2.2.2. The Pluriseptate Varieties of *Arthopyrenia persoonii* Treated as *Arthopyrenia grisea*

*Arthopyrenia persoonii* was established by Massalongo [11] in response to the heterogeneous historical use of the name *Verrucaria punctiformis* Pers. Massalongo emphasized the high variability of the taxon and recognized several varieties within *A. persoonii*, noting that some might ultimately deserve recognition at species level on the basis of their combined characters. He described eight varieties, distinguished by differences in thallus morphology, ascomatal characters and ascospore morphology. Several of these, including var. *juglandis*, var. *mali*, var. *pancina*, and var. *scotina*, were characterized by pluriloculate ascospores (mostly > 3 septate). Among these, var. *scotina* was described as a comparatively distinct morphotype, with larger, confluent ascomata and larger spores, and is therefore not included in the detailed comparison below.

*Arthopyrenia persoonii* var. *juglandis*, var. *mali*, and var. *pancina* are primarily distinguished by ascospore morphology and ascus shape (Table 1). Var. *juglandis* has relatively broad, ellipsoid-clavate ascospores (12.2–18.3 × c. 6.1 µm) with 3–5 transverse septa and broad clavate asci; Massalongo himself regarded this taxon as potentially distinct at the species level. Var. *mali* differs in having longer and distinctly narrower, clavate ascospores with 5–7 transverse septa and elongate-clavate asci. Var. *pancina* also produces 5–7-septate, ellipsoid-clavate ascospores, but differs from var. *mali* in its slightly broader spores; its ascomata are small, subhemispherical and commonly bear a minute apical papilla.

**Figure 5:**
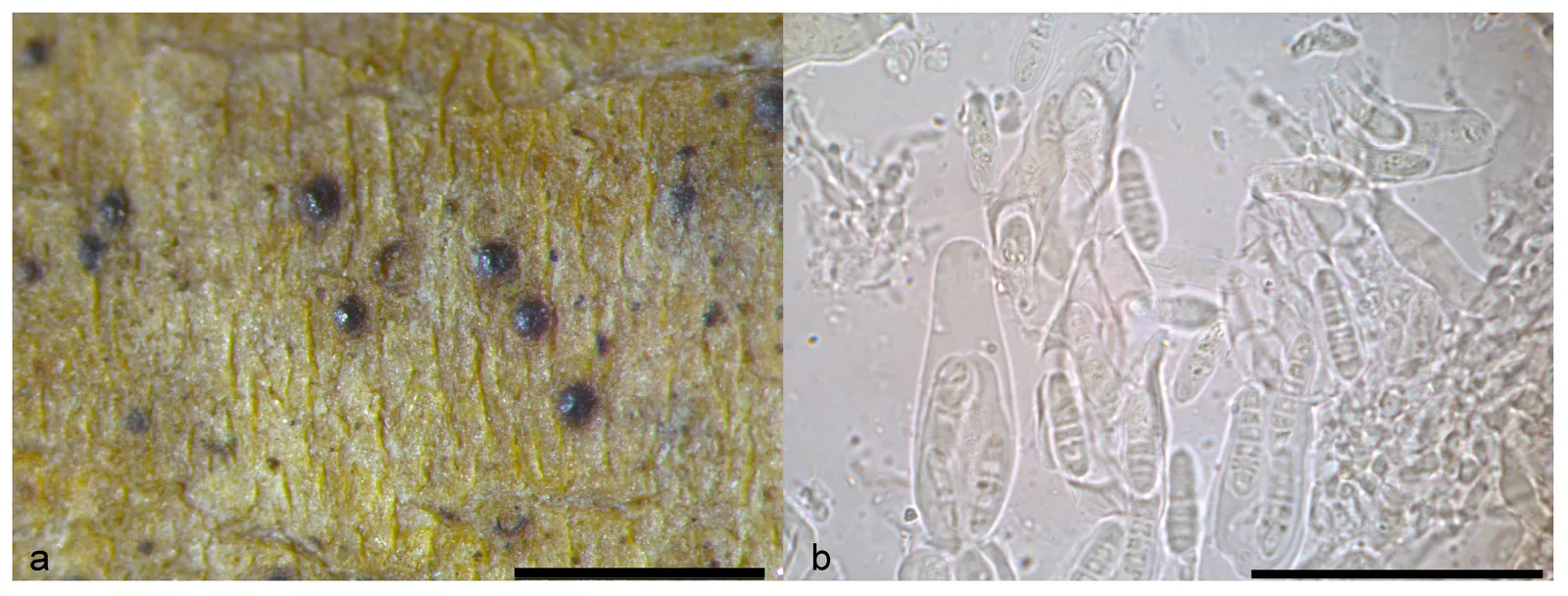
*Lectotype of Arthopyrenia persoonii* var. *juglandis* A. Massal. (**a**) Enlarged view of the thallus showing numerous small, dark ascomata; scale bar = 1 mm. (**b**) Bitunicate ellipsoid-clavate asci and 3–5-septate ascospores in 10% KOH; scale bar = 50 µm.

**Figure 6:**
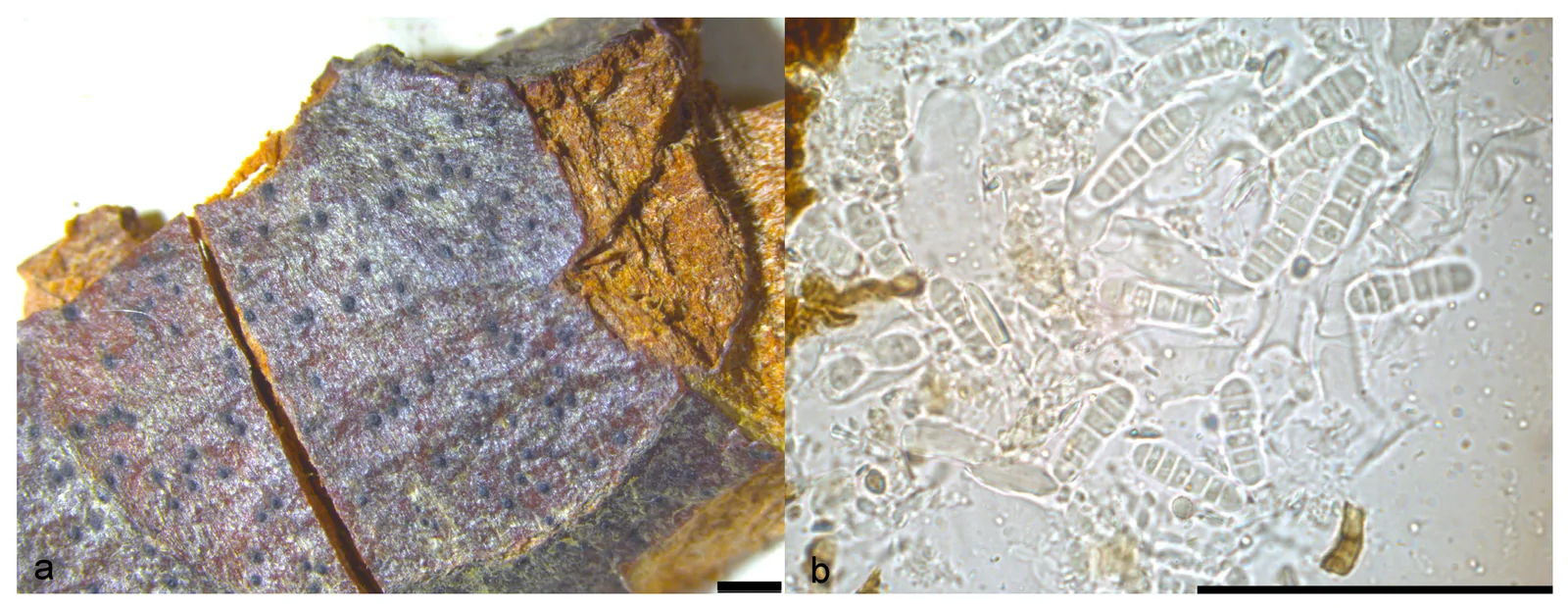
*Lectotype of Arthopyrenia persoonii* var. *mali* A. Massal. (**a**) Enlarged view of one of the bark fragments of the exsiccatum showing an extremely thin, immersed in the bark thallus; scale bar = 1 mm. (**b**) 5–7-septate ascospores in 10% KOH; scale bar = 50 µm.

**Figure 7:**
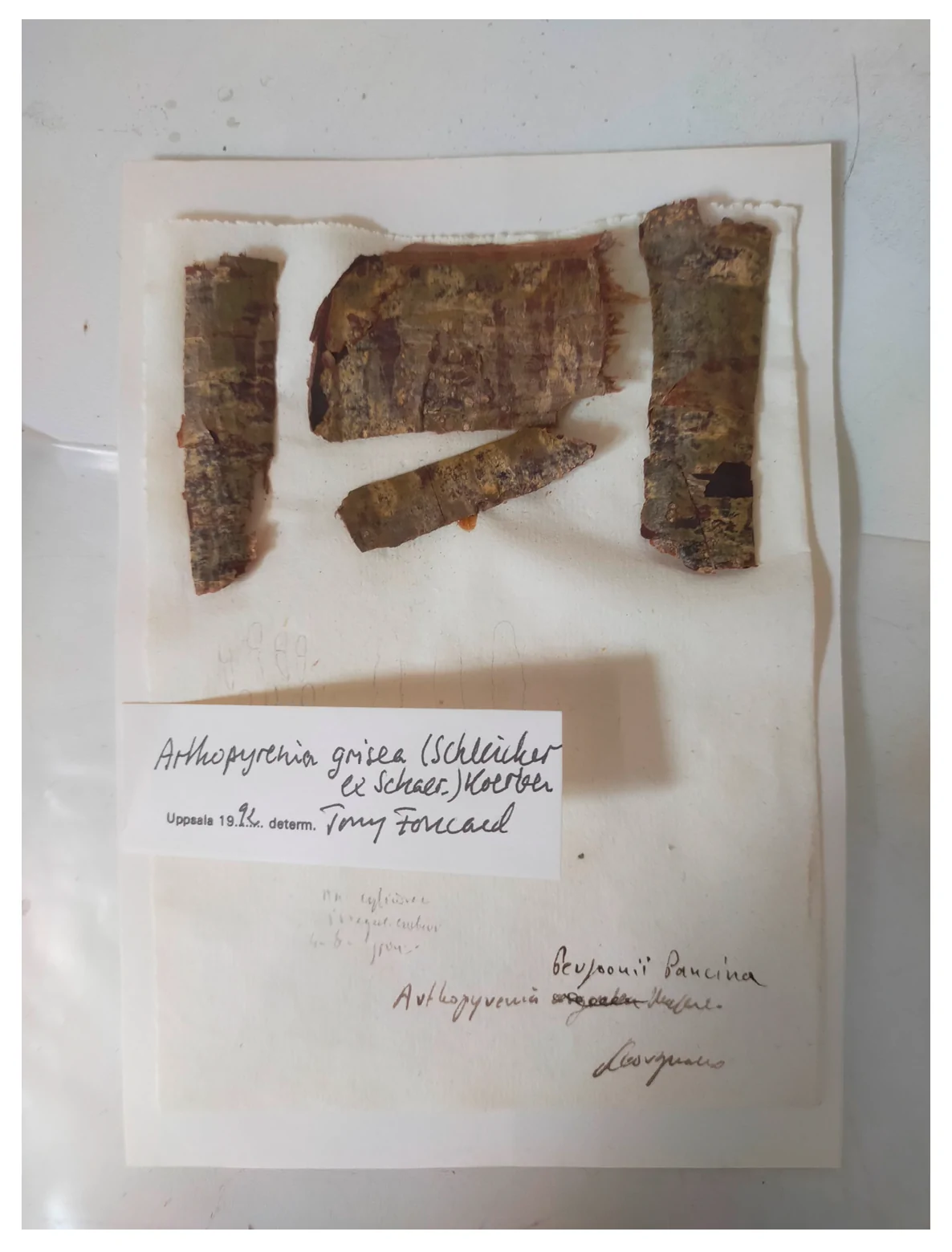
Lectotype of *Arthopyrenia persoonii* var. *pancina* A. Massal.

Foucard [5] subsequently examined original material of the *A. persoonii* varieties *juglandis* (Figure 5), *mali* (Figure 6), and *pancina* (Figure 7) preserved at VER. He considered specimens referred to *A. persoonii* and *A. pluriseptata* (Nyl.) Arnold to be conspecific with *A. grisea* (Schaer.) Körb., the latter name having nomenclatural priority. The relationship between *A. molinii* and *A. pluriseptata* is particularly relevant because Arnold considered the two taxa scarcely distinct at species level [12]. However, the name *A. pluriseptata* has been applied in different senses in the historical literature, making direct comparison dependent on the material and species concept involved. Lazzarin [6] subsequently dealt with type material of several Massalongo names preserved in the A.B. Massalongo Herbarium at VER. The lectotypes of the three varieties examined here were designated and were therefore not re-typified in the present study. Their morphology was reassessed and compared with the respective protologues.

The pluriloculate varieties of *A. persoonii* treated as *A. grisea* [5], differ from *A. molinii* in having an extremely thin thallus immersed in the bark and lacking a conspicuous black hypothallus (Figure 6a). Their perithecia are mostly scattered, not surrounded by dark areoles (Figure 5a), and smaller (0.15–0.20 vs. 0.18–0.24 mm in diam.). Although the ascospores are likewise pluriseptate (3–5 in var. *juglandis*, 5–7 in var. *mali* and *pancina*) (Figure 5b and Figure 6b), they are clavate to ellipsoid-clavate, and generally smaller compared with the larger, narrowly clavate to obclavate, 21–23 × 4–5 µm, 5–7-septate ascospores of *A. molinii* (Figure 2b).

Re-examination of the original material confirms that the three varieties are morphologically differentiated, supporting Massalongo’s recognition of distinct varietal forms. However, the available material is insufficient to determine whether these morphological differences correspond to discrete evolutionary lineages.

#### 2.2.3. *Arthopyrenia platypyrenia* (Nyl.) Arnold

*Arthopyrenia platypyrenia* is a western European species, with rare and scattered records from Ireland, Great Britain, France, and Italy, mostly collected on *Hedera helix* L. [13]. In Italy, this species is known only from Calabria [14] and Tuscany (Pisa), where it has recently been collected on *Pittosporum tobira* (Thunb.) W.T. [15]. It differs from *A. molinii* in having a thinner, less conspicuous thallus that does not form distinct tartareous patches, and larger, more flattened perithecia lacking the dark areolas typical of *A. molinii*. The hymenium also differs in containing well-developed persistent pseudoparaphyses. In addition, the ascospores are typically ellipsoidal to fusiform-ellipsoidal, 3–4(–7)-septate, distinctly constricted at all septa and surrounded by a gelatinous sheath 2–3 µm thick; they are also larger (24–30 × 8–10 µm) than those observed in *A. molinii*.

## 3. Discussion

### Arthopyrenia molinii and Related Pluriseptate Taxa

The historical treatment of the pluriseptate *Arthopyrenia* complex illustrates the difficulties involved in delimiting taxa known primarily from historical material and interpreted under different species concepts. The recurrent association of *A. molinii* with *A. pluriseptata*, *A. parolinii*, and pluriseptate forms of *A. persoonii* reflects the broad morphological similarity among taxa sharing pluriseptate ascospores, but also differences in the weight assigned to other characters.

Arnold, in his treatment of *A. pluriseptata*, explicitly stated that *A. molinii* and *A. parolinii* were “vix specifice diversae”, thereby regarding them as scarcely distinct at species level in relation to *A. pluriseptata* [12]. The latter was characterized by an externally inconspicuous thallus, small ascomata, nearly absent paraphyses, and elongate-oblong, 5–7-septate ascospores. In contrast, the material examined in the present study shows that *A. molinii* possesses a persistent and conspicuous epiphloeodal thallus, relatively large perithecia, often associated with dark basal areoles, and narrowly clavate to narrowly obclavate ascospores. These differences indicate that the similarity between the taxa is largely confined to a subset of characters, most notably ascospore septation, rather than extending to their overall morphological combination.

A similar issue applies to the pluriseptate varieties of *A. persoonii* treated as *A. grisea* [5]. Their re-examination confirms that these forms are morphologically differentiated from *A. molinii*, despite sharing a comparable degree of ascospore septation. This supports the importance of retaining a multicharacter approach when evaluating historical taxa.

The comparison with *A. parolinii* and *A. platypyrenia* reinforces this interpretation. Although both taxa, like *A. molinii*, has pluriseptate ascospores, their overall morphological combinations differ, indicating that pluriseptation is better regarded as a character shared among several taxa than as evidence of close taxonomic affinity by itself.

Taken together, these observations support the recognition of *A. molinii* as a distinct morphological entity on the basis of the historical material currently available. However, this interpretation remains provisional because the species is known exclusively from historical collections. In the absence of recent collections and molecular data, it is not possible to assess the extent of morphological variation within *A. molinii* or to determine whether the morphological entities recognized here correspond to independently evolving lineages.

## 4. Materials and Methods

The study was based on examination of the protologue of *Arthopyrenia molinii* Beltr. [8], identification of the locus classicus, and a systematic search for original material. The search was conducted in European herbaria and was based on the name published in the protologue, the unpublished name *Arthopyrenia summana* Beltr., the localities and substrate reported in the protologue, historical annotations, herbarium provenance, and exsiccata labels. Specimens were considered original material when they could be demonstrably connected with the material used by Beltramini for the description of the species.

Original material was investigated through direct examination and, where direct access was not possible, through high-resolution digital images provided by the respective herbaria. Historical specimens from M (Botanische Staatssammlung München), RO (Herbarium of Sapienza University of Rome), and VER (Herbarium A.B. Massalongo, Civic Natural History Museum of Verona), selected as the lectotype, were examined directly, both macroscopically and microscopically. Additional specimens were examined from high-resolution digital images provided by BM/NHMUK (Natural History Museum, London), K (Royal Botanic Gardens, Kew), M, NY (New York Botanical Garden), and VE (Museo di Storia Naturale Giancarlo Ligabue of Venezia). Additional occurrence records retrieved through the Global Biodiversity Information Facility GBIF were used to trace further historical material (GBIF Occurrence Download, available online: https://doi.org/10.15468/dl.rpw45g; accessed on 3 September 2026); records for which suitable specimen images or sufficient information for morphological examination were unavailable were not considered for morphological assessment.

Macromorphological features were examined on dried material using a Zeiss stereomicroscope (Carl Zeiss Microscopy, Jena, Germany) equipped with a Leica digital camera (Leica Microsystems GmbH, Wetzlar, Germany). Micromorphological characters were studied from hand-cut sections and squash preparations mounted in water (H_2_O) and 10% potassium hydroxide (KOH; Sigma-Aldrich, St. Louis, MO, USA), using a Nikon Eclipse Si light microscope equipped with a Digital Sight 10 microscope camera (Nikon Corporation, Tokyo, Japan). Anatomical characters of the thallus, ascomata, ascomata wall, involucrellum, excipulum, hamathecium, asci, and ascospores were recorded. Amyloid reactions were tested using Lugol’s iodine solution (I; Sigma-Aldrich, St. Louis, MO, USA). Measurements were taken from structures observed in the lectotype and are reported as ranges; additional specimens were examined for comparison.

Historical specimens of selected varieties of *A. persoonii* and related taxa were investigated using the same criteria and procedures described above. A complete list of the historical specimens examined, including herbarium repositories, accession numbers, type status, and method of examination, is provided in Appendix A (Table A1).

The selection of the lectotype was based on the availability of original material, its correspondence with the protologue, the information provided by the associated labels, and the degree to which the specimen allowed the morphological characters and provenance of the name to be documented.

Nomenclatural decisions and typification follow the International Code of Nomenclature for algae, fungi and plants [16].

## 5. Conclusions

The present study lectotypifies *A. molinii* and provides a provisional morphological circumscription based on historical material. The species is characterized by the combination of an epiphloeodal thallus, relatively large perithecia frequently associated with dark basal areoles, and narrow, 5–7-septate ascospores. These features distinguish it from the other pluriseptate taxa examined here, but the absence of recent collections precludes assessment of intraspecific variation and of the stability of these characters. Because no molecular data are available and most of the taxa considered here has been reliably recollected in recent decades, the generic and species-level placement of A.* molinii* must remain provisional. Targeted searches in the type localities and in ecologically similar habitats are required to test whether these historical names correspond to extant, distinguishable lineages.

## Figures and Tables

**Table 1:** Comparative morphological reassessment of the three pluriloculate varieties of *Arthopyrenia persoonii* Massal. based on historical material and original descriptions.

Character	var. *juglandis*	var. *mali*	var. *pancina*
Thallus	Extremely thin, light brown, tending towards tawny brown, immersed in the bark	Extremely thin, dark greyish-brown, immersed in the bark, inconspicuous	Very thin, inconspicuous, immersed in the bark
Perithecia	Very small (ca. 0.10 mm diam.), immersed, scattered, flattened-hemispherical to subelliptic, depressed around the ostiole	Small (ca. 0.15–0.20 mm diam.), immersed, scattered, rarely confluent, hemispherical, depressed around the ostiole	Very small (<0.10 mm diam.), scattered, subhemispherical and subpapillate at the apex
Asci	Clavate, up to twice as wide as in related varieties.	Clavate-cylindrical, of standard width for the taxon	Clavate-cylindrical
Spore septation	3–5-septate	5–7-septate	5–7-septate
Spore size	12.2–18.3 × ca. 6.1 µm	≥18.3 × ca. 3.7 µm	ca. 18.5 × 4 µm
Spore shape	Clavate to ellipsoid-clavate, distinctly broader	Clavate to ellipsoid-clavate, markedly elongated and slender	Ellipsoid-clavate
Typical substrate	*Juglans regia* L.	*Malus* spp.	Not substrate-specific

## Data Availability

The data supporting the findings of this study are available within the article and its Appendix A. The GBIF occurrence data used in this study are publicly available through the GBIF Occurrence Download (https://doi.org/10.15468/dl.rpw45g).

## Appendix A

This appendix provides a complete list of the historical specimens examined for *A. molinii* and related taxa, including herbarium repository, specimen or accession number, nomenclatural status, and method of examination. Specimens were assessed as original material where their provenance and associated historical information allowed them to be linked to the material on which the relevant name was based. Both specimens examined directly and those studied from high-resolution digital images are included.

**Table A1:** Historical specimens of *Arthopyrenia molinii* and related taxa examined in the present study, including herbarium repositories, specimen or accession numbers, type status, and method of examination.

Taxon	Herbarium	Specimen Number	Material	Method of Examination
*A. molinii*	BM/NHMUK	BOT BM001107632	Uncertain	Digital images
	K	K-M000969962	Uncertain	Digital images
	M	M-0207337/630042/287563	Uncertain	Direct
	M	M-0207338/630043/287564	Uncertain	Digital images
	M	M-0207339/630044/287565	Uncertain	Digital images
	NY	04633650	Uncertain	Digital images
	RO	H.C.24.I.8, sheet 34	Uncertain	Direct
	VER	AM4491	Original	Direct
*A. parolinii*	M	M-0207340	Uncertain	Direct
	VE	MSNVE-24815	Not original	Direct
	VER		Lectotype	Direct
*A. persoonii* var. *juglandis*	RO	70.II.4., sheet 6	Uncertain	Direct
	TO	TO0018302	Not original	Direct
	VE	MSNVE-25624	Uncertain	Digital images
	VER	liAM58	Lectotype	Direct
	VER	LIE 256	Not original	Direct
*A. persoonii* var. *pancina*	RO	H.R.63.I.2, sheet 95	Not original	Direct
	TO	TO0019869	Not original	Direct
	VE	MSNVE-25623	Original	Digital images
	VER	LIE 254 B	Uncertain	Direct
*A. persoonii* var. *mali*	RO	70.II.4., sheet 8	Not original	Direct
	VER	liAM44	Lectotype	Direct
	VER	LIE 257	Not original	Direct
*A. platypyrenia*	CLU	8069	Not original	Direct
	PI	PI 067248	Not original	Direct
